# Correction: Xiang, S., *et al.* Schisandrin B Induces Apoptosis and Cell Cycle Arrest of Gallbladder Cancer Cells. *Molecules* 2014, *19*, 13235–13250

**DOI:** 10.3390/molecules21050673

**Published:** 2016-05-20

**Authors:** Shan-Shan Xiang, Xu-An Wang, Huai-Feng Li, Yi-Jun Shu, Run-Fa Bao, Fei Zhang, Yang Cao, Yuan-Yuan Ye, Hao Weng, Wen-Guang Wu, Jia-Sheng Mu, Xiang-Song Wu, Mao-Lan Li, Yun-Ping Hu, Lin Jiang, Zhu-Jun Tan, Wei Lu, Feng Liu, Ying-Bin Liu

**Affiliations:** 1 Department of General Surgery, Xinhua Hospital, Affiliated to Shanghai Jiao Tong University School of Medicine, No. 1665 Kongjiang Road, Shanghai 200092, China; 2 Laboratory of General Surgery, Xinhua Hospital, Affiliated to Shanghai Jiao Tong University School of Medicine, No. 1665 Kongjiang Road, Shanghai 200092, China; 3 Institute of Biliary Tract Disease, Shanghai Jiao Tong University School of Medicine, No. 227 South Chongqing Road, Shanghai 200025, China; 4 The First Affiliated Hospital Nanchang University Emergency Unit, No. 17 Yongwai Road, Nanchang 330006, China

We would like to change the Affiliation addresses on Page 13235 of paper [1], from:

1 Department of General Surgery, School of Medicine, Shanghai Jiao Tong University, No. 1665 Kongjiang Road, Shanghai 200092, China
2 Laboratory of General Surgery, School of Medicine, Xinhua Hospital, Affiliated to Shanghai JiaoTong University, No. 1665 Kongjiang Road, Shanghai 200092, China
3 Institute of Biliary Tract Disease, School of Medicine, Xinhua Hospital, Affiliated to Shanghai JiaoTong University, No. 1665 Kongjiang Road, Shanghai 200092, China

to the correct version, as follows:

1 Department of General Surgery, Xinhua Hospital, Affiliated to Shanghai Jiao Tong University School of Medicine, No. 1665 Kongjiang Road, Shanghai 200092, China
2 Laboratory of General Surgery, Xinhua Hospital, Affiliated to Shanghai Jiao Tong University School of Medicine, No. 1665 Kongjiang Road, Shanghai 200092, China
3 Institute of Biliary Tract Disease, Shanghai Jiao Tong University School of Medicine, No. 227 South Chongqing Road, Shanghai 200025, China

We apologize for any inconvenience brought to the readers.
